# Risk Mapping of Groundwater‐Drawdown‐Induced Land Subsidence in Heterogeneous Soils on Large Areas

**DOI:** 10.1111/risa.12890

**Published:** 2017-10-30

**Authors:** Jonas Sundell, Ezra Haaf, Tommy Norberg, Claes Alén, Mats Karlsson, Lars Rosén

**Affiliations:** ^1^ Department of Architecture and Civil Engineering Chalmers University of Technology Gothenburg Sweden; ^2^ COWI AB Gothenburg Sweden; ^3^ Department of Earth Sciences University of Gothenburg Gothenburg Sweden; ^4^ Department of Mathematical Sciences Chalmers University of Technology and the University of Gothenburg Gothenburg Sweden

**Keywords:** Groundwater‐drawdown‐induced subsidence, probabilistic subsidence model, risk‐based decisions, risk communication, sensitivity analysis

## Abstract

Groundwater leakage into subsurface constructions can cause reduction of pore pressure and subsidence in clay deposits, even at large distances from the location of the construction. The potential cost of damage is substantial, particularly in urban areas. The large‐scale process also implies heterogeneous soil conditions that cannot be described in complete detail, which causes a need for estimating uncertainty of subsidence with probabilistic methods. In this study, the risk for subsidence is estimated by coupling two probabilistic models, a geostatistics‐based soil stratification model with a subsidence model. Statistical analyses of stratification and soil properties are inputs into the models. The results include spatially explicit probabilistic estimates of subsidence magnitude and sensitivities of included model parameters. From these, areas with significant risk for subsidence are distinguished from low‐risk areas. The efficiency and usefulness of this modeling approach as a tool for communication to stakeholders, decision support for prioritization of risk‐reducing measures, and identification of the need for further investigations and monitoring are demonstrated with a case study of a planned tunnel in Stockholm.

## 1. INTRODUCTION

Groundwater‐drawdown‐induced subsidence due to leakage of groundwater into subsurface constructions or overextraction of groundwater is a severe problem in many regions around the world, including Shanghai,^1^ Mexico City,^2^ Bangkok,^3^ Las Vegas,^4^ and the Scandinavian cities such as Stockholm, Gothenburg, and Oslo.^5^, ^6^ In areas with compressible soil deposits, a groundwater drawdown will induce consolidation settlements that potentially damage buildings and other constructions. Since a groundwater drawdown often implies a large area of influence, see, e.g., Burbey^4^ and Huang *et al*.,^7^ the potential damage cost in an urban areas is substantial.^8^

**Figure 1:**
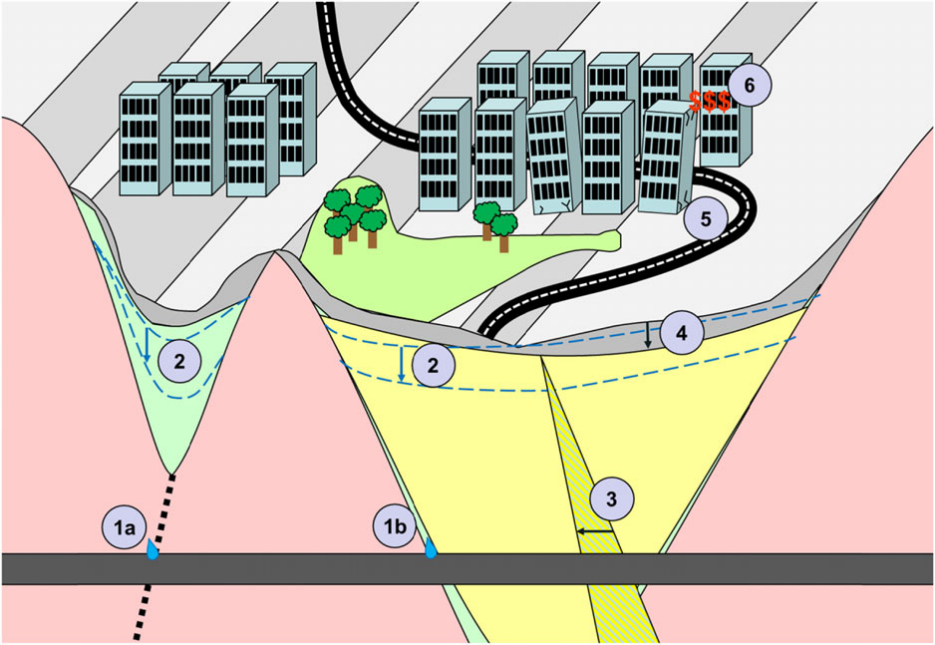
The chain for groundwater‐drawdown‐induced subsidence damages and its included processes. The pink area illustrates bedrock; green: coarse‐grained material; yellow: soft clay; and gray: coarse‐grained filling material. The hatched line at (a) illustrates a fracture zone in the bedrock, modified from Sundell.^10^

**Figure 2:**
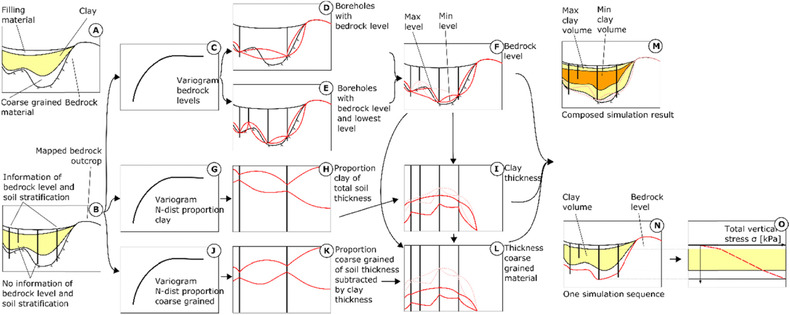
Simulation sequence for soil stratification and total vertical stress.

The cause–effect chain of groundwater‐drawdown‐induced subsidence (Fig. 1) is initiated with groundwater extraction due to pumping or leakage of groundwater into a subsurface construction in bedrock (Fig. 1a) or soil (Fig. 2b). It continues with the reduction of groundwater,^2^ pore pressure reduction in compressible deposits,^3^ and subsidence.^4^ The sensitivity of the constructions founded on the compressible deposits determines the extent of the subsidence damages.^5^ Finally, the economic consequences depend on the cost^6^ associated with the damage.

The suggested method in this study is limited to the third (pore pressure) and the fourth (subsidence) part of the chain. For a risk assessment of the whole cause–effect chain, the method needs to be combined with estimations of groundwater‐drawdown magnitudes, sensitivity of constructions founded on the compressible deposits, and costs associated with the potential subsidence damages.^9^, ^10^

The large spatial scale of the problem domain implies heterogeneous soil conditions formed by geological processes and anthropogenic activities such as construction, excavation, filling of material, and groundwater drawdowns. Therefore, the subsurface cannot be described in detail without extensive and costly site investigation programs. Commonly, only limited information is available, necessitating characterization of uncertainties in the heterogeneous conditions. In urban settings, large numbers of borehole logs describing soil stratigraphy from previous construction projects are commonly available.^11^, ^12^, ^13^ On the other hand, records of analyzed soil samples are less frequent. For a case study in Stockholm (see Section 3.), 20,000 borehole logs of soil stratification stand against 79 piston samples in clay with evaluated material properties.

Depending on the heterogeneity of the soil, available information, and sampling density, appropriate methods should be used for uncertainty estimation. In cases with sufficient spatial density of the samples, the geostatistical interpolation method kriging^14^ is a useful tool for the estimation of spatial uncertainty. Kriging is based on a variogram that describes the relationship between the mean variance between paired data values separated by a lag distance, see, e.g., Marache *et al*.^12^ According to a literature review by Li *et al*.,^15^ the distance for which geotechnical properties of clay are correlated typically fall within the ranges of 10–90 m (horizontally) and 0.1–8.0 m (vertically). An analysis of clay samples from a site in Sweden found horizontal correlation ranges of up to about 30 m.^16^ Beyond the correlation range, the best estimate of a particular property corresponds to the mean of the probability density function (PDF) for all samples of that property.

Several probabilistic methods exist for prediction of land subsidence. Griffiths and Fenton^17^ demonstrate a method based on random field estimation of permeability properties applied in a finite‐element model. However, this method does not take variability in soil stratification into account and is not applied on a large scale. Ryu *et al*.^18^ use indicator kriging for the simulation of random fields for both soil stratification and compression parameters and calculate subsidence with a one‐dimensional (1D) model. Although this method is applied on a large scale, it does not account for dependencies between the parameters and trends along depth for compression parameters. Marache *et al*.^19^ use a finite‐element model and kriging for uncertainty estimation of soil stratification and compression parameters. Even though this publication presents a model for soil stratification on a city scale, soil properties and subsidence are only modeled on a local scale (0.4 km²).

The large scale of the problem, the heterogeneity of the soil, and the typically dense data sets for soil stratigraphy contrasted by sparse data for soil properties call for a novel probabilistic modeling approach that is capable of combining these different sources of information. The aim of this study is to present a spatial probabilistic method for simulation of groundwater‐drawdown‐induced subsidence on a macroscale (1–100 km²) with a simple 1D compression model. The simulation is based on a statistical analysis of soil stratification and compression parameters. Uncertainty and heterogeneity in soil stratification is quantified with kriging, whereas uncertainties in compression parameters are based on a statistical analysis that takes into account vertical trends and dependencies between the compression parameters. When these two methods are combined in a subsidence simulation, the result gives a dense spatial resolution of land subsidence risks over large areas. Applied on a case study for a planned tunnel in Stockholm, Sweden, the result of the simulation is mapped as risk areas. Together with other information, such as estimation of extent and magnitude of groundwater drawdown and sensitivity of risk objects, the risk areas provide decision support when planning for further investigations, safety measures, and monitoring.

Pumping or leakage ratio can vary between construction and operation phase, which also affects the reduction of pore pressure over time. A delimitation of this study is that we do not consider the evolution of settlements over time but only the somewhat conservative scenario of final settlements. Therefore, the suggested method should be used for studying situations with permanent or long‐term drainage of groundwater.

The structure of the article is as follows: Section 2. presents methodology and assumptions for simulation of soil stratification, reduction of pore pressure, and subsidence. Section 3. introduces the Stockholm case study with results for simulation and sensitivity analysis. Finally, conclusions are presented in Section 4..

## 2. METHOD

This section describes the suggested method and assumptions for probabilistic subsidence modeling on large areas, which includes a probabilistic soil stratification model, assumptions of pore pressure and its decrease with depth due to groundwater drawdown, the 1D nonlinear compression model, data processing, and statistical analysis of compression parameters as well as simulation of subsidence.

### 2.1. Soil Stratification Model and Vertical Stress

A geological model provides information on continuity of stratigraphy between boreholes and helps to understand spatial variation. The simulation of soil stratification follows the procedure presented in Sundell *et al*.^20^ but a short summary applied on simulation of vertical stress follows here. For the case study, the soil stratification is conceptualized into three continuous layers: filling material, clay, and coarse‐grained glacial material (glacial till and/or glaciofluvial deposits) on top of the bedrock; see (Fig. 2(A)). Since not all boreholes contain all necessary information to build a soil stratification model, the method is based on a stepwise procedure to consider all data and dependencies between layers. The following information is included in the boreholes (Fig. 2(B)): bedrock levels, lowest level without reaching bedrock, and interpreted soil layers. Mapped bedrock outcrops are also included in the simulations.

In a first step, a variogram is modeled from boreholes with bedrock levels (Fig. 2(C)). From kriging of boreholes with bedrock levels, grids with average levels and standard deviations are used for simulation (min‐ and max‐levels in Fig. 2(D)). Since continuous variables with infinite support are assumed in kriging, the min and max levels correspond to a low and a high quantile such as the 5th and 95th percentile. Similarly, bedrock levels and the lowest level from boreholes where bedrock is not detected are simulated (min‐ and max‐levels in Fig. 2(E)). From Figs. 2(D) and (E), the same quantile from each simulation is compared and the lowest level is selected as the resulting simulated bedrock level (Fig. 2(F)). Due to this process, boreholes without detected bedrock but with a deep lowest level, close to the bedrock, are considered in the model, whereas boreholes without bedrock but with a high lowest level close to the ground surface are left out of the model.

From boreholes with information on soil stratification, the parameter *z_pb_* is defined as the proportion of clay, calculated and transformed to a normal distribution from the probability integral of the standardized normal distribution *N*(0,1). This step is necessary to make the simulation of clay independent from the previous bedrock simulation. From these transformed values, a variogram is modeled (Fig. 2(G)). As for the bedrock levels, the kriging gives expected values and standard deviations, which are used for simulation of proportion of clay thickness out of total soil thickness (min and max values shown in Fig. 2(H)). When the bedrock is simulated (Fig. 2(F)), the resulting soil thickness is multiplied with the simulated proportion of clay thickness (Fig. 2(H)), which gives the clay thickness (min and max values in Fig. 2(I)).

To simulate the vertical location of the clay layer in the soil profile, the proportion of coarse‐grained material out of the total soil thickness subtracted with the clay thickness is calculated and transformed to normality with the previous mentioned method (introduces parameter *z_pc_*). The clay thickness has to be subtracted for an independent simulation of coarse‐grained material. From these transformed values, a variogram is modeled (Fig. 2(J)), and the proportions are simulated (Fig. 2(K)). Based on the result of the total soil thickness (from simulation of bedrock) and clay thickness, the thickness of the coarse‐grained material is simulated (Fig. 2(L)).

The result of Figs. 2(F), (I), and (L) gives a simulation of the soil profile. Fig. 2(N) gives an example of one simulation sequence and Fig. 2(M) presents max and min values of clay thickness from composed simulation results. In each simulation of soil stratification, the total vertical stress (*σ*) is calculated from the unit weight of the materials (Fig. 2(O)). Since data sets with borehole logs are often located closely, soil stratigraphy and vertical stress can often be simulated with a relatively high spatial resolution. For the case study in Section 3., a horizontal grid with 10 × 10 m resolution, resulting in a total of 130,000 vertical vectors at all grid points, is chosen for interpolations and simulations. This 10‐m resolution is sufficiently detailed to both cover individual risk objects and to describe the heterogeneity of the soil conditions. The vertical resolution of each vector is chosen to be 0.1 m.

### 2.2. Pore Pressure and Effective Stress

**Figure 3:**
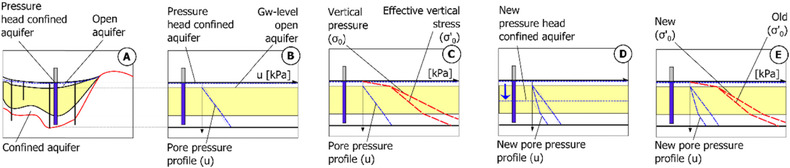
One simulation sequence of pore pressure (B), effective vertical stress (C), new pressure head due to groundwater drawdown in confined aquifer (D), and new effective stress (E).

Pore pressure conditions in a soil profile depend on the hydraulic conductivity of the materials, drainage length, and water balance between infiltration and drainage in the different layers. Although reduction of pore pressure due to groundwater drawdown is a transient process, this study is limited to study the steady‐state conditions at the end of the process. If detailed measurements of pore pressure (*u*) are not present, estimations are made by assuming a linear dependency between the open and the confined layers, see, e.g., Persson^8^ and Zeitoun and Wakshal.^21^ Since a clay layer has a very low hydraulic conductivity, hydrostatic conditions between the open and the confined aquifer (Fig. 3) cannot be assumed. In the case study, pressure heads in the confined aquifer are obtained from an interpolation of average levels from 300 groundwater observation wells. Since observations of heads in the open aquifer are few, groundwater levels in this layer are assumed to correspond to the top of the clay layer.^22^, ^23^ Assuming higher groundwater heads (closer to ground surface) in the open aquifer would result in greater pore pressure (*u*) and lower effective vertical stress (*σ′*_0_). Such assumptions thus result in a higher overconsolidation ratio (OCR) and a greater preconsolidation margin (*σ′_v_‐σ′*_0_), which means that the critical plastic phase in stage 2 (see Section 2.3.) is less likely to occur. Therefore, the given assumption is conservative since it results in greater subsidence magnitudes than the assumption of a higher groundwater head in the open layer. Based on these conditions, the pore pressure varies according to a straight line between the pressure at the bottom and the top of the clay layer (Fig. 3(B)).

The deformation of a saturated granular medium containing water within its voids is governed by the effective stress *(σ′*_0_). Here, the effective stress (*σ’*_0_), i.e., the intergranular load distribution, is the total stress (*σ*) minus the pore pressure (*u*) (Fig. 3(C)). When a groundwater drawdown occurs in the confined aquifer, the pore pressure is reduced (Fig. 3(D)), which also results in a new effective (vertical) stress (Fig. 3(E)). In the case study, pore pressure reductions and changes in effective stress corresponding to 0.5, 1, and 2 m of groundwater drawdown in the confined aquifer are calculated at each grid point. As with *σ*, *u* and *σ′*_0_ are simulated at each of the vertical grid points and at every 0.1‐m interval.

### 2.3. Compression Parameters and Subsidence Model

Consolidation settlement in soft soils occurs as a result of change in effective stress. To calculate subsidence, we use a simple, but in Sweden well‐established, 1D elasto‐plastic model by Larsson and Sällfors.^24^ The model is based on parameters evaluated from constant rate of strain (CRS) oedometer tested clay samples. This model is in accordance with the well‐recognized concept of stress–strain behavior of clays under 1D straining by Bjerrum,^25^ the guidelines for evaluation of parameters from oedometer compressibility in Eurocode 7,^26^, ^27^, ^28^ and common practice in geotechnical engineering; see, e.g., Fang.^29^ In addition, the CRS method is similar to the international ASTM standard for *One‐Dimensional Consolidation Properties of Saturated Cohesive Soils Using Controlled Strain Loading*.^30^ As mentioned in Pu and Fox,^31^ the CRS test has several advantages compared to the standard incremental loading oedometer test, including generation of a continuous stress–strain curve and a shorter test period. Disadvantages of the CRS test include inability to evaluate creep (secondary or delayed consolidation)^24^ and possible dependence between the measured response and the applied strain rate.^31^

**Figure 4:**
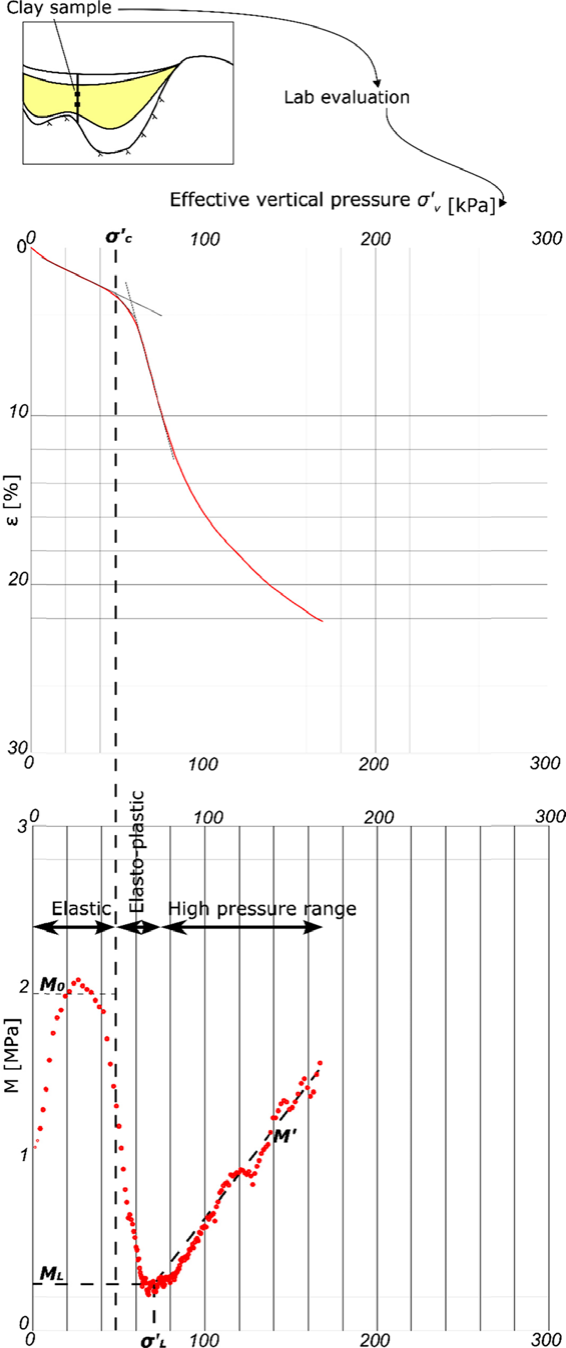
Example of results from a CRS test and evaluated parameters σ′*_c_*, σ′*_L_*, *M_L_*, *M*_0_, and *M*′ from a clay sample together with the three stiffness regimes.

Similar to Janbu's tangent modulus approach,^32^ soil compressibility is evaluated from a diagram where vertical strain (*ε*) is plotted versus effective vertical stress (*σ′*); see Fig. 4. As with the more recognized compression index method, see e.g., Fang 29, different parameters are estimated for the normal and the overconsolidated part of the curve. Comparisons and transformations between the different methods can be found in Refs. 33, 34, 35. There are more advanced models that both consider creep and anisotropy of the clay properties, see, e.g., Sivasithamparam *et al*.^36^ and Olsson,^37^ but the chosen model is detailed enough to capture the dominant modes of response in the system.

The subsidence calculation method is based on the evaluation of compression parameters from piston samples of clay evaluated at a CRS of 0.7% per hour. Three stiffness regimes with different modulus of compressibility (*M*) as function of *σ′* are evaluated (Fig. 4):

(1) The initial stage in the consolidation process is considered linear elastic. As discussed in, e.g., Fang^29^ and Olsson,^35^ the methods for estimating the modulus *M*_0_ in this stage give results with significant uncertainties. For the case study in this article, the modulus *M*_0_ is evaluated with the empirical relationship based on undrained shear strength from fall cone tests, *τ_fu_*, *M*_0_ ≈ 250*τ_fu_ as suggested by Larsson *et al*.^38^ and Moritz.^39^
(2) When a material yields, it goes from elastic to elasto‐plastic conditions. If only 1D conditions are considered, the transition phase from elastic to yielding is often simplified to a yield point, corresponding to the preconsolidation stress *(σ′_c_)*. In Sweden, the industry standard for estimating the preconsolidation stress follows a graphical method introduced by Sällfors;^40^ see hatched lines in Fig. 4. When elasto‐plastic conditions are reached after the yield point, the strain increases and plastic hardening occurs, increasing the preconsolidation stress. At *σ′_c_*, the modulus is assumed to drop constantly to the *second constant modulus*, *M_L_*. Since the stiffness after preconsolidation is significantly smaller, i.e., leading to larger subsidence magnitudes, accurate estimation of preconsolidation stress is critical.
(3) At higher stresses, the assumption of a constant modulus ends and a third phase occurs (stress > *σ′_L_* in Fig. 4) with a constantly increasing modulus. At this part of the curve, the modulus number *M′* is evaluated as Δ*M*/*σ′*.^24^

Fig. 4 presents how the compression parameters *σ′_c_, σ′_L_*, *M_L_*, *M*_0_, and *M′* correspond to the evaluated curve for a sample at a certain depth below surface. To calculate subsidence, compression parameters are needed for the whole soil profile. If several samples are taken at the same location but on different depths, the parameter result should be interpolated or divided into representative segments. Depending on the effective stress and its change (Δ*u* = Δ*σ* in this case) along a soil profile, the three phases are related to corresponding equations for the calculation of subsidence (Equations (1)–(3) in Table I). Equation (4) integrates the result from the different segments.

**Table I:** Equations (1)–(4) for Calculation of Final Subsidence According to Larsson and Sällfors^24^

	Case	Equations
Equation (1)	$\sigma_{o}^{\prime }+\Delta \sigma <\sigma_{c}^{\prime }$	$\delta \;\left(z\right)=\frac{\Delta \sigma }{M_{0}}\;$
Equation (2)	$\sigma_{c}^{\prime }<\sigma_{o}^{\prime }+\Delta \sigma <\sigma_{L}^{\prime }$	$\delta \;\left(z\right)=\left(\frac{\sigma_{c}^{\prime }-\sigma_{0}^{{}^{\prime }}}{M_{0}}+\frac{\sigma_{0}^{{}^{\prime }}+\Delta \sigma -\sigma_{c}^{{}^{\prime }}}{M_{L}}\right)$
Equation (3)	$\sigma_{o}^{\prime }+\Delta \sigma >\sigma_{L}^{\prime }$	δ(z)=σc′−σ0′M0+σL′−σc′ML+1M′ ln 1+(σ0′+Δσ−σL′)M′ML
Equation (4)	–	s=∫0z max δ(z)dz

*Note*: Equation (4) integrates the total subsidence for each segment.

### 2.4. Data Processing and Statistical Analysis of Parameters

Before defining PDFs for Monte Carlo (MC) simulation of subsidence, spatial variations and dependencies between the parameters need to be addressed.

#### 2.4.1. Dependencies Between Parameters

**Figure 5:**
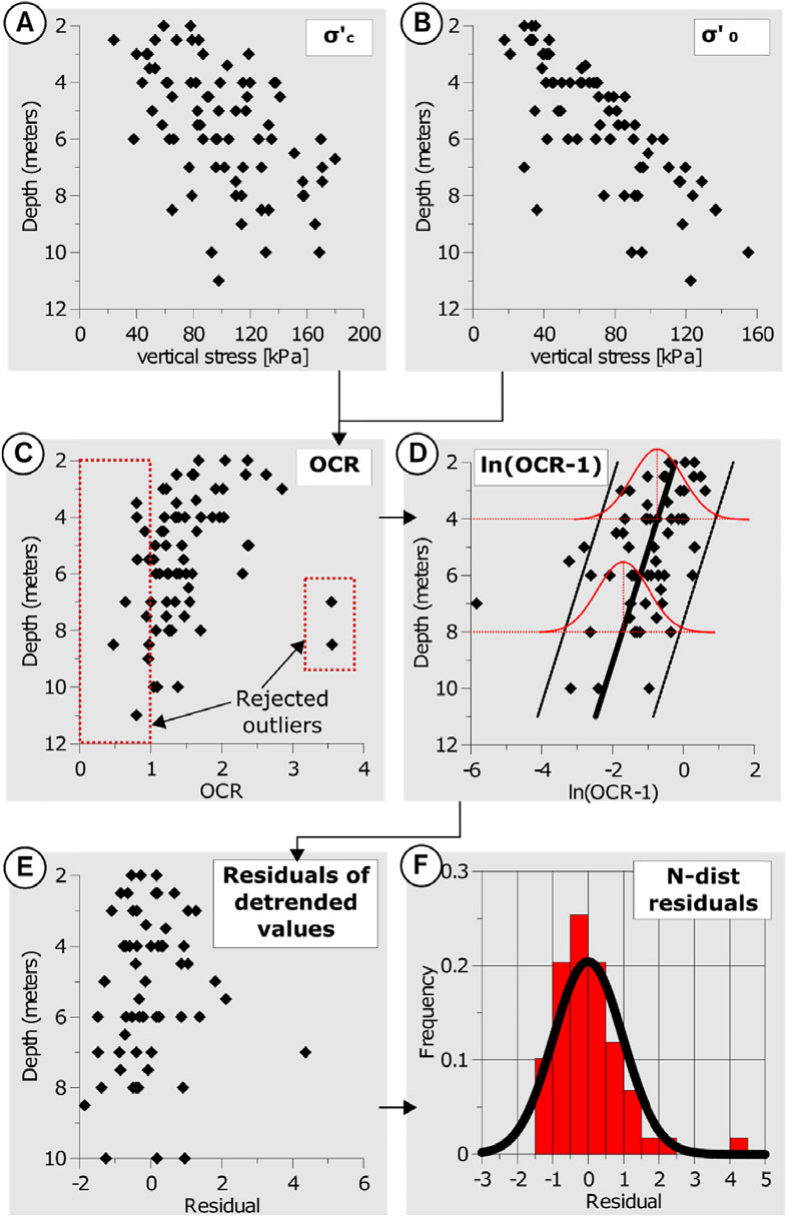
Process of transforming data to control dependencies between parameters. The illustration is exemplified with the dependency between *σ*′_0_ and *σ*′_c_.

Before PDFs for simulations can be constructed, it has to be confirmed that the variables are independent. First, the dependency between the previously calculated current effective stress, *σ′*_0_ (see Section 2.2.) and preconsolidation stress, *σ′_c_* (Figs. 5(A) and (B)) is investigated. The relationship between these parameters defines the OCR (*σ′_c_*/*σ′*_0_) (Fig. 5(C)). *σ′*_0_ cannot be higher than *σ′_c_* since *σ′_c_* represents the maximum value of an historical effective stress unless the soil is in the process of consolidating from a previous applied load. This condition results in a dependency between *σ′*_0_ and *σ′_c_* with the condition OCR > 1. With this condition, all OCR values <1 have to be carefully evaluated. Reasons to reject these values for further analysis include disturbances during sampling, lab evaluation, overestimated pore pressure (*u*), and/or underestimated total stress (*σ*). Rejected outliers are marked with two red boxes in Fig. 5(C); see Section 3.3. for details.

After identified outliers have been eliminated, the data are ln‐transformed to normality (Fig. 5(D)). Before this step, the OCR values are subtracted by 1 in order to condition the subsequent simulation to OCR > 1. The logarithm of the values is taken to assure homoscedastic errors (equal variance at each vertical interval, exemplified on depth 4 and 8 m along the regression line in Fig. 5(D)). Possible vertical trends are investigated with linear regression (Fig. 5(D)) resulting in residuals (Fig. 5(E)) represented by a normal distribution (Fig. 5(F)); see, e.g., Tang^41^ and Lacasse and Nadim^42^ for similar transformations of OCR.

Other dependencies exist as a consequence of the stress–strain relationship (Fig. 4). To preserve these dependencies, two criteria are introduced: *σ′_L_* > *σ′_c_* and *M*_0_ > *M_L_*. Analogous to *σ′_c_* and OCR, inconsistencies in subsequent simulations are avoided by scaling with the ratios *σ′_L_*,/*σ′_c_* and *M*_0_/*M_L_*. As with OCR, *σ′_L_*,/*σ′_c_* is first subtracted with 1 to condition *σ′_L_* > *σ′_c_* in subsequent simulations. As a final step, the parameter ratios are transformed and possible vertical trends are investigated (analogous with the example for OCR in Fig. 5).

In addition, dependencies between *σ′_L_* and *M_L_* are investigated with the same procedure since these parameters are related in the stress–strain curve.

**Figure 6:**
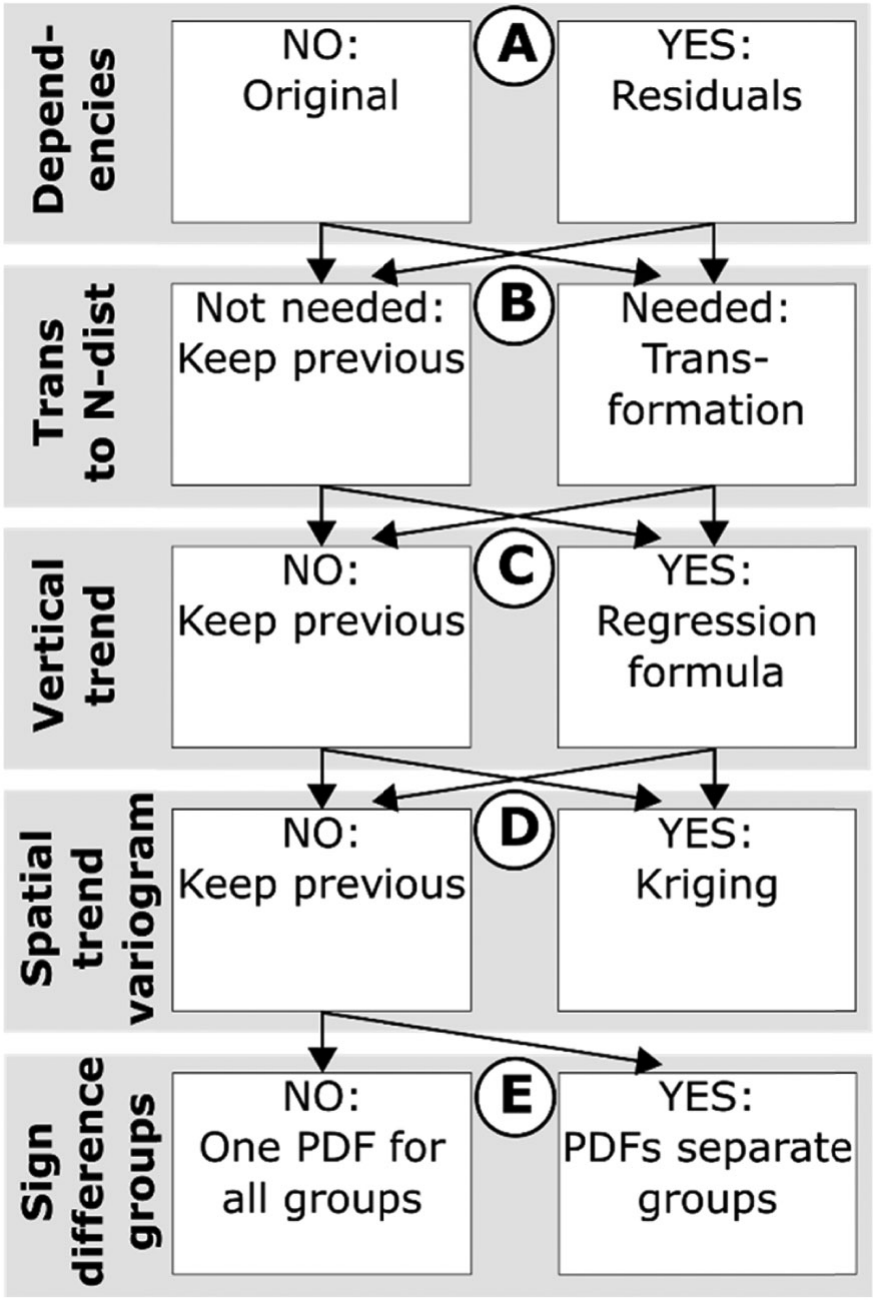
Scheme for data processing and analysis before probability density functions (PDFs) are generated.

The necessary steps for a successful data analysis and distribution fitting are summarized in Figs. 6(A)–(C). In Fig. 6(A), dependencies are investigated, such that if a parameter is dependent of another, the parameter ratio is used. In Fig. 6(B), the data are transformed to normality. If the data from a previous step already can be described with a normal distribution, transformation is unnecessary. Finally, in Fig. 6(C), vertical trends are investigated with linear regression. When *R* ^2^ is close to zero, absence of a vertical trend can be assumed. However, if trend partly describes parameter uncertainties (low *R* ^2^), ignoring the trend will result in an overestimation of the spread in the sample, hence an overestimation of uncertainties in subsequent simulations. For these cases, the residuals between the regression line and the data are used in subsequent steps. As a final stage, the data are tested for normality with residual and normal‐score plots together with the Kolmogorov–Smirnov test; which measures the supremum of the pointwise distances between data and fitted distribution; see, e.g., Johnson *et al*.^43^

#### 2.4.2. Spatial Variations

Spatial correlation in the horizontal plane is investigated with two methods: variogram analysis (as with the boreholes for soil stratification in Section 2.1.) and analysis of variance (ANOVA). Before these methods can be used, two requirements need to be fulfilled: (1) the data are normally distributed with equal variances and (2) there is no vertical trend in the data. If the data do not meet these conditions, the transformations in Section 2.4.1. (Figs. 6(A)–(C)) are applied.

If the variograms can reveal a significant spatial trend (Fig. 6(D)), kriging is used to simulate a field of average values and standard deviations. Unlike boreholes describing soil stratification, soil samples are often spatially scarce, which means that the correlation range in the variogram is likely to be shorter than the distance between the samples; If this is the case, differences between groups of samples can be investigated with ANOVA; see, e.g., Marx and Larsen.^44^ In the case study, two different group divisions are evaluated based on the following assumptions: (1) clay from the same valley represents the same sedimentological unit and has similar properties, and (2) the load history is different between clay sampled in a heavily constructed area compared to a greenfield site (degree of urbanization, DU). The division between the two groups is presented in the Appendix. The null hypotheses (equal means among groups) are rejected at the significance level of 0.05. If the null hypothesis can be rejected, the Bonferroni method^45^ is used as a *post hoc* test to compare differences between means. If the result of the *post hoc* test reveals significant differences between groups (at the 5% level), these groups are used to define PDFs in the subsequent step (Fig. 6(E)). If the null hypothesis cannot be rejected, differences between the groups cannot be distinguished and all samples are assumed to belong to the same population.

#### 2.4.3. PDFs

Depending on the results in previous steps, two different methods are used to generate PDFs. The first method is applied on both cases for the final step in Fig. 6. With this method, PDFs are constructed with the *t*‐distribution from the sample mean, standard deviation, and degrees of freedom; see, e.g., Marx and Larsen.^44^ If the number of data points is large, the normal distribution can be used instead. Fig. 5(F) illustrates how the residuals of ln(OCR − 1) follow a normal distribution.

If the variogram analysis reveals significant spatial variations, kriging is used to model a grid with average values and standard deviations at every interpolation point as with the soil stratification model in Section 2.1..

### 2.5. Simulation of Subsidence

**Figure 7:**
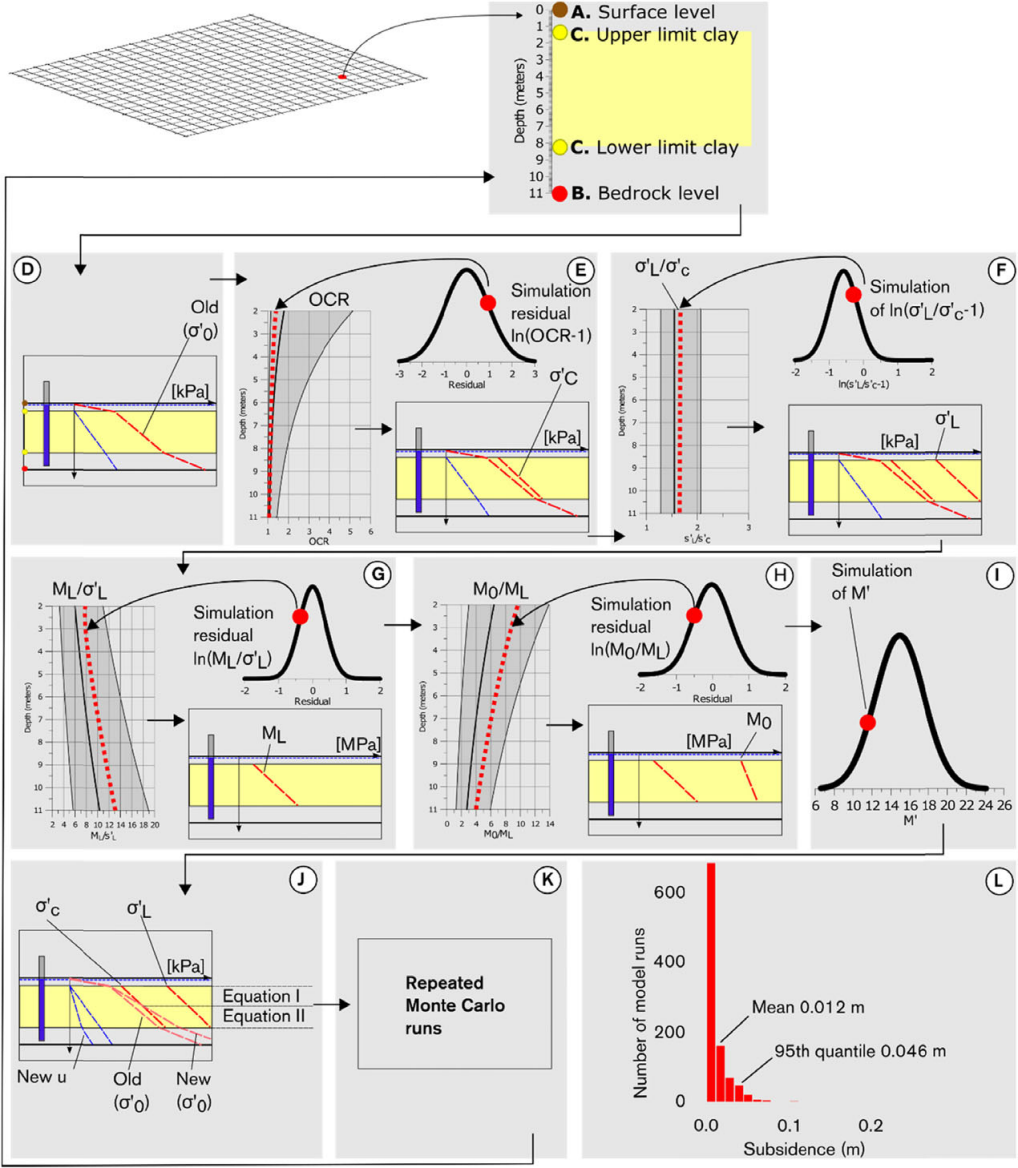
Simulation sequence for compression parameters and subsidence.

Based on the previous described simulation of soil stratification, effective stress, and the PDFs of the parameters, subsidence is simulated with a MC model. The simulation sequence is illustrated in Fig. 7. The spatial resolution of the simulation is 10 × 10 m, consistent with the soil stratification model. Although different variants of data processing and generation of PDFs are possible for each parameter, as suggested in Section 2.4.3., each step is represented by the applied model in the Stockholm case study; see Section 3.3..

At each grid point, the simulation is initiated with a bedrock level (Fig. 7(B)) and soil stratification (Fig. 7(C)) according to the procedure presented in Section 2.1.. At each 0.1‐m depth increment, *σ′*_0_ (before groundwater drawdown) is calculated (Fig. 7(D)) according to the description in Section 2.2..

In the next step (Fig. 7(E)), *σ′_c_* is calculated by multiplying OCR with the previous simulated *σ′*_0_. OCR is calculated by first simulating a value from a PDF describing residuals of ln(OCR − 1). From the difference between the average regression line and the simulated residual, values for ln(OCR − 1) are calculated at each vertical interval. These values are transformed to values of OCR through the natural exponential function and by adding 1. The shaded area in the regression graph in Fig. 7(E) illustrates the 90% confidence limits for observations of OCR. The red hatched line illustrates a simulation of OCR based on the exemplified iteration from the PDF for the residual of ln(OCR − 1).

From *σ′_c_* and a PDF for ln(*σ′_L_*/*σ′_c_* − 1) values for *σ′_L_* are simulated (Fig. 7(F)). Similarly as with OCR, *M_L_* is calculated from iterations from a PDF describing the residuals of ln(*M_L_*/*σ′_L_*), a regression line for ln(*M_L_*/*σ′_L_*), and the previously simulated *σ′_L_* (Fig. 7(G)). Subsequently, *M*_0_ is calculated in the same manner but based on the simulated value of *M_L_*, a simulated residual for ln(*M*_0_/*M_L_*), and its regression line (Fig. 7(H)). The only parameter that is independent of other parameters and depth is *M′*, which is simulated from a normal distribution (Fig. 7(I)).

From the previous calculation of change in effective stress (Figs. 3(E) and 7(J)), the updated effective stress *σ′*_0_ due to groundwater drawdown is compared with *σ′_c_* and *σ′_L_* and the adequate Equation (1)–(3) in Table I is selected. From the selected equation and the simulated parameters, subsidence is calculated for each iteration. In the example in Fig. 7(J), Equation (1) is used for the top part where *σ′*_0_ + Δ*σ* < *σ′_c_*, meanwhile Equation (2) is used for the bottom of the soil profile where *σ′_c_* < *σ′*_0_ + Δ*σ* < *σ′_L_*. Subsidence is calculated at each 0.1‐m interval and total subsidence approximated at each grid point with the trapezoidal rule (Equation (4)).

The whole simulation sequence is repeated with 1,000 iterations at each grid point (Fig. 7(K)). From these iterations, a resulting distribution of subsidence magnitudes is obtained (Fig. 7(L)). By combining the result distributions of all grid points, the subsidence risk is mapped spatially; see Sections 3.4. and 3.5..

## 3. STOCKHOLM CASE STUDY

This section presents the case study with data, statistical analysis of the data, simulation results, sensitivity analysis, and risk mapping.

### 3.1. Study Area

The method is applied to a case, the City Link tunnel in Stockholm, which is a planned utility tunnel in bedrock for power lines. The study area in Stockholm (59°19′N 18°4′E) is located on the East Coast of Sweden and covers approximately 15 km² (Fig. 11). The geology in the area consists of several valleys in pre‐Cambrian crystalline bedrock, partly filled with glaciofluvial sediments such as sand, gravel, and clay. Since the tunnel will be constructed in crystalline bedrock, significant subsidence due to tunnel deformation can be dismissed. The soil stratification is simplified to three distinct layers: (1) postglacial sand, filling material, or organic deposits; (2) glacial and postglacial clay; and (3) coarse‐grained glacial material (glaciofluvial sand, glacial till) above the bedrock surface. The study area is delimited by a conservative estimate of maximum extent for groundwater drawdown in the confined aquifer due to leakage of groundwater into the planned tunnel.

### 3.2. Sampling Strategy

To find a representation of the geotechnical properties of the clay, three criteria were set defining the spatial target areas for sampling: (1) within clay‐covered valleys where thick layers of clay could be expected; (2) within a predicted influence area for groundwater drawdown; and (3) where constructions sensitive for subsidence damages are located within (1) and (2). A total of 79 piston samples from 38 locations were taken during year 2013–2014; see location of samples in Fig. 11 and result of parameter estimation in the Appendix. The sampling procedure followed Swedish standard practice.^46^ Other field tests such as cone penetration tests, static sounding, and soil/rock drilling were executed in conjunction with the piston samples.

In addition, about 20,000 boreholes containing information on soil stratification exist from previous construction projects. Out of these, 14,300 are selected for modeling and the rest used as a validation data set; see Sundell *et al*.^20^ From the modeling data set, 6,500 boreholes contain information on bedrock levels, 7,800 do not reach the bedrock, and 4,000 contain information on soil stratification.

### 3.3. Data Processing and Statistical Analysis of Samples

**Figure 8:**
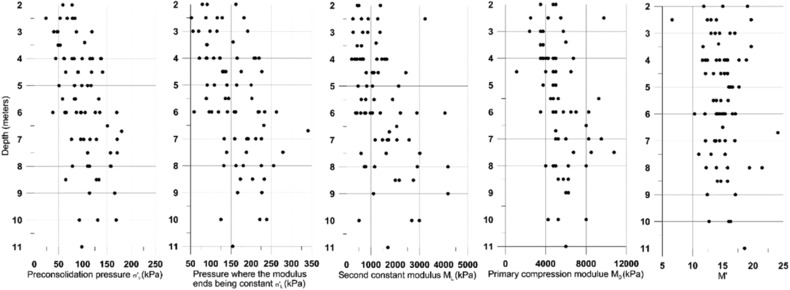
Scatter plots of *σ*′*_c_*, *σ*′*_L_*, *M_L_*, *M*_0_, and *M*′ from the 79 samples.

The compression parameters from the 79 samples in the case study are plotted against depth in Fig. 8. As seen from the figure, all samples except for *M′* reveal a small trend with higher values along depth.

According to the procedure in Section 2.4.1., dependencies are accounted for through OCR − 1, *σ′_L_*/*σ′_c_* − 1, and *M*_0_/*M_L_*. Out of the 79 samples, 72 contains all information needed to calculate *σ′*_0_ and OCR. Eleven samples are rejected due to an OCR < 1. These rejected values are likely due to disturbances during sampling, lab evaluation, an overestimated pore pressure (*u*), and/or an underestimated total stress (*σ*). Outliers for two samples with an OCR > 3 are also rejected; see Fig. 5. These values are likely a result of an underestimated groundwater level at the location for these samples in point 13C323; see the Appendix. Although the rejections indicate low sampling quality, these uncertainties cannot be reduced without additional investigations with higher‐precision methods. In addition to the above‐mentioned dependencies, ln(*σ′_L_*) and ln(*M_L_*) reveal a moderately strong linear dependency (*R* ^2^ = 0.63). To take account of this dependency, the ratio *M_L_*/*σ′_L_*, is introduced. No dependency between other parameters and *M′* was found. *M′* is therefore kept in its original form.

**Figure 9:**
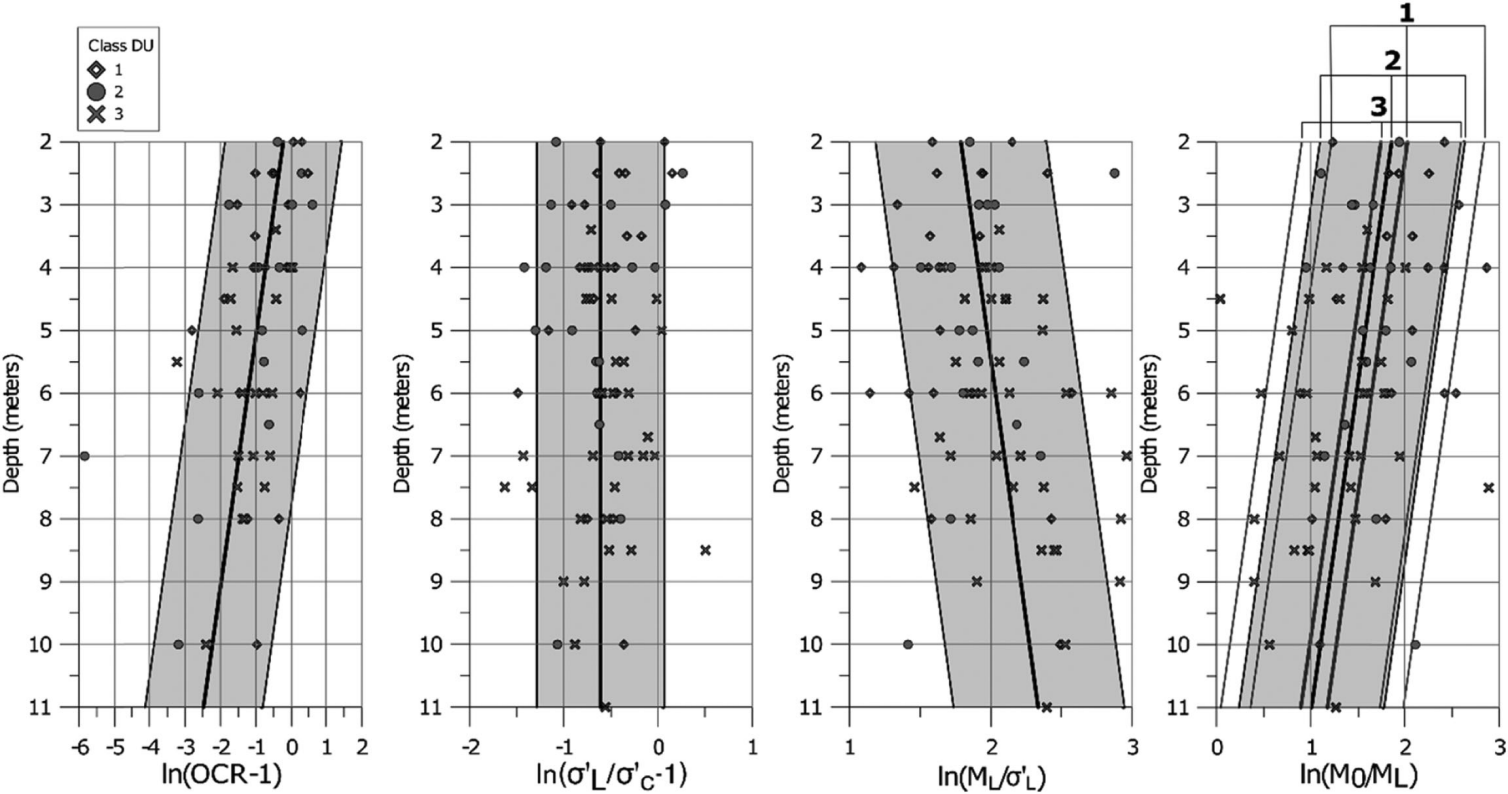
Transformed compression parameters along depth with regression line (thick) and 90% confidence interval for future observations (shaded). Since no significant correlation was detected for ln(*σ*′*_L_*/*σ*′*_c_* − 1), the regression line is substituted with a line representing the mean value. The outlier value of ln(OCR − 1) = −6 is due to an observation of OCR close to 1.

In the next step, OCR − 1, *σ′_L_*/*σ′_c_* − 1, *M*_0_/*M_L_*, and *M_L_*/*σ′_L_* are transformed to normality with the natural logarithm. Possible vertical trends are investigated with linear regression, *y* = *ax* + *b*, where *y* is the parameter value (plotted on the horizontal axis in Fig. 9), *a* is the slope, *x* is the independent variable (depth below ground surface), and *b* is the intercept in Table II. Three parameters, ln(OCR − 1), ln(*M_L_*/*σ′_L_*), and ln(*M*_0_/*M_L_*), reveals a trend that cannot be neglected (*R*² > 0.1) since it partly describes parameter uncertainties. Two parameters, ln(*σ′_L_*/*σ′_c_* − 1) and *M′* show no significant trend, which means that the regression result is ignored in the next step. The regression lines for ln(OCR − 1), ln(*M_L_*/*σ′_L_*), and ln(*M*_0_/*M_L_*) are presented in Fig. 9.

**Table II:** Regression Coefficients and Coefficients of Determination (*R*²) for the Transformed Parameters

Parameter ‐ *y*	ln(OCR − 1)	ln(*σ*′*_L_*/*σ*′*_c_* − 1)	*M*′	ln(*M_L_*/*σ*′*_L_*)	ln(*M*_0_/*M_L_*)
Coefficient of determination ‐ *R* ^2^	0.22	0.01	0.01	0.10	0.13
Intercept ‐ *b*	0.27	−0.48	0.61	1.67	2.05
Slope ‐ *a*	−0.25	−0.02	–	0.06	−0.10

The transformed parameters (residuals of ln(OCR − 1), residuals of ln(*M_L_*/*σ′_L_*), residuals of ln(*M*_0_/*M_L_*), ln(*σ′_L_*/*σ′_c_* − 1), and *M′*) are tested for normality with residual and normal‐score plots together with the Kolmogorov–Smirnov test; see, e.g., Johnson *et al*.^43^ For the Kolmogorov–Smirnov test, all parameters have an observed significance level greater than 0.05, thus indicating normality. The residual and normal‐score plots result in homogeneous scatters and no significant deviations from the ideal line, which indicates a successful transformation to normality.

In the tests for spatial variability according to Section 2.4.2., the variogram analysis reveals no spatial correlation. This is likely due to the large distance (>100 m for most cases) between the samples; see variograms in the Appendix. In the ANOVA test, the null hypothesis cannot be rejected for residuals of ln(OCR − 1), residuals of ln(*M_L_*/*σ′_L_*), ln(*σ′_L_*/*σ′_c_* − 1), and *M′* in either of the two group divisions (see the Appendix). As no spatial correlations are detected for these parameters, all samples are considered to belong to the same population. Consequently, the complete data set is used to form PDFs for subsidence predictions at all locations. Since the sample size is large, means and standard deviations according to Table III describe normally distributed PDFs. Based on the standard deviations, the 90% intervals for future observations are illustrated by the shaded areas in Fig. 9.

**Table III:** Mean and Standard Deviations of Parameters Forming Normally Distributed PDFs for MC Simulation

Parameter	Mean	*SD*
Residual ln(OCR − 1)	0	1.00
ln(*σ*′*_L_*/*σ*′*_c_* − 1)	−0.61	0.41
Residual ln(*M_L_*/*σ*′*_L_*)	0	0.37
DU1 Residual ln(*M*_0_/*M_L_*)	−0.17	0.49
DU2 Residual ln(*M*_0_/*M_L_*)	0	0.47
DU3 Residual ln(*M*_0_/*M_L_*)	0.11	0.51
*M*′	14.93	2.60

For the residuals of ln(*M*_0_/*M_L_*), the null hypothesis is rejected for both group divisions. The *post hoc* Bonferroni test results in a significant difference only between DU subgroups 1 and 3 and no significant difference between any subgroups in the other group division of samples close to each other. As a result of this difference, the residuals of ln(*M*_0_/*M_L_*) are grouped accordingly: DU1 includes samples from groups 1 and 2, DU2 includes 1, 2, and 3, and DU3 groups 2 and 3. With this group division, normally distributed PDFs with mean values and standard deviations according to Table III are created. The average deviation from the regression line and the 90% interval for future observations are illustrated by the top most numbers 1, 2, 3 of the regression graph for ln(*M*_0_/*M_L_*) in Fig. 9. Since DU2 includes all samples, there is no deviation between this group and the original regression line. Although a significance difference exists between the three subgroups, this difference is not very large, as seen in Fig. 9. Since the PDFs for the three subgroups are significantly overlapping, the practical difference in simulation between the different DU groups and the significance of a possible type I error is therefore of minor importance. Nevertheless, the three different group divisions for the residuals of ln(*M*_0_/*M_L_*) are used in the subsequent simulation stage. Similarly for the cases where the null hypothesis cannot be rejected in the case of a type II error, a division between groups would result in significantly overlapping PDFs, causing only minor difference in the calculated subsidence magnitudes. A complete evaluation of compression parameters with ANOVA is presented in Ramm and Collinder.^47^

For calculation of vertical stress together with the simulation of soil profile, the density of clay and the coarse‐grained material are evaluated from samples. The results are represented by PDFs N(19;0.9) and N(17.5;0.9) kN/m³, respectively.

### 3.4. Simulation of Subsidence

Based on the PDFs of the parameters described in Table III and the simulation scheme in Section 2.5., subsidence is simulated for each of the groundwater drawdown magnitudes of 0.5 m, 1 m, and 2 m in the confined aquifer. The spatial deviation of the DU areas for simulation of respective residual for ln(*M*_0_/*M_L_*) are presented on a map in the Appendix. The whole simulation process takes approximately 50 hours on a current‐generation PC workstation.

**Figure 10:**
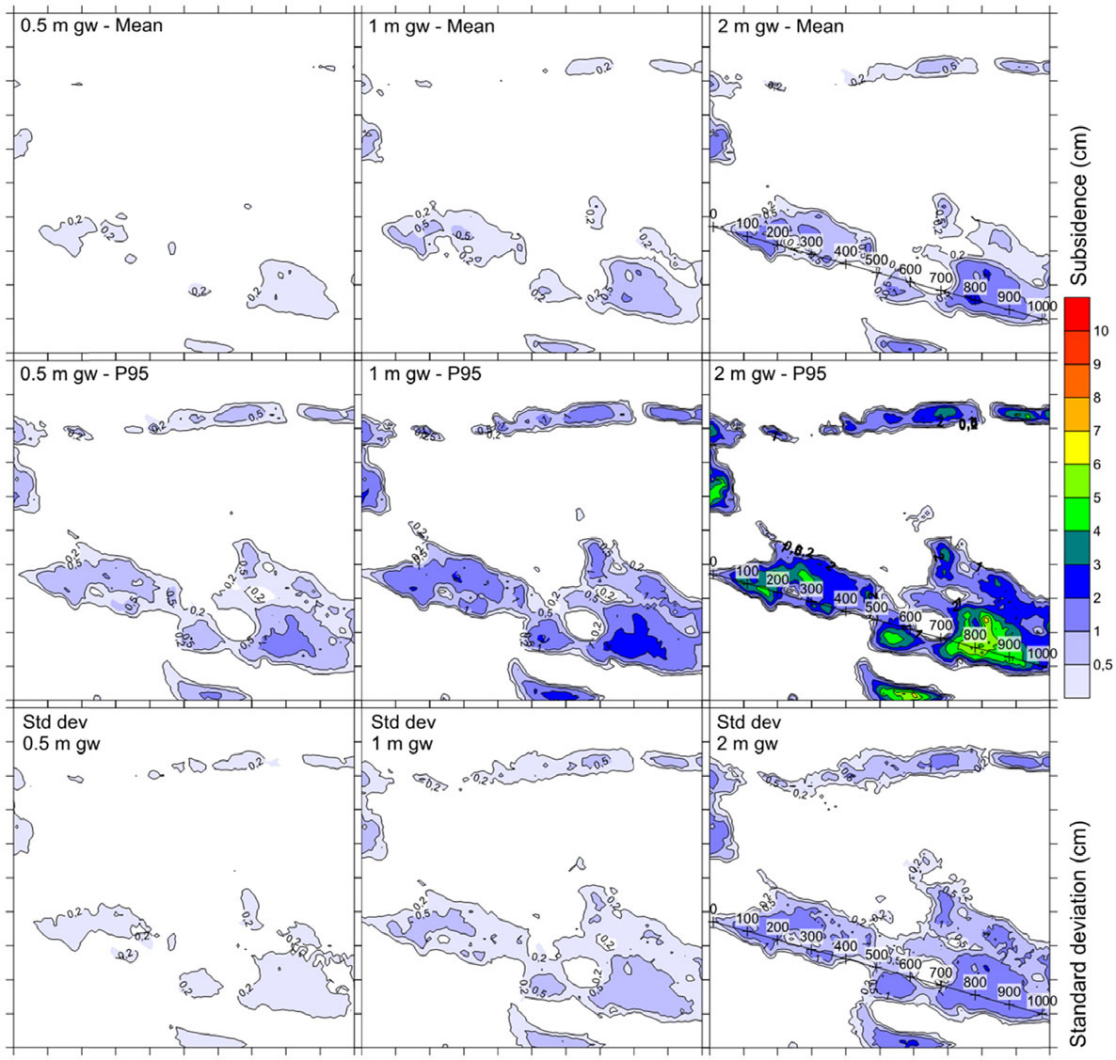
Mean value, 95th percentile, and standard deviation of the subsidence simulations for the three levels of groundwater drawdown (0.5, 1, 2 m). The figure covers an outcrop of the total area shown in Fig. 11. The tick marks on the *x*‐ and *y*‐axis are on 100 m distance. The numbered line shows the location for the cross‐section in Fig. 13.

From the result distributions, mean values, 95th percentiles, and standard deviations are presented in Fig. 10. To simplify presentation, only a detail of the total study area is shown. As seen from the figure, there is a significant difference between the calculated mean value and the 95th percentile for each of the drawdown scenarios. There is also a noteworthy difference between the different drawdown magnitudes.

### 3.5. Risk Map

**Figure 11:**
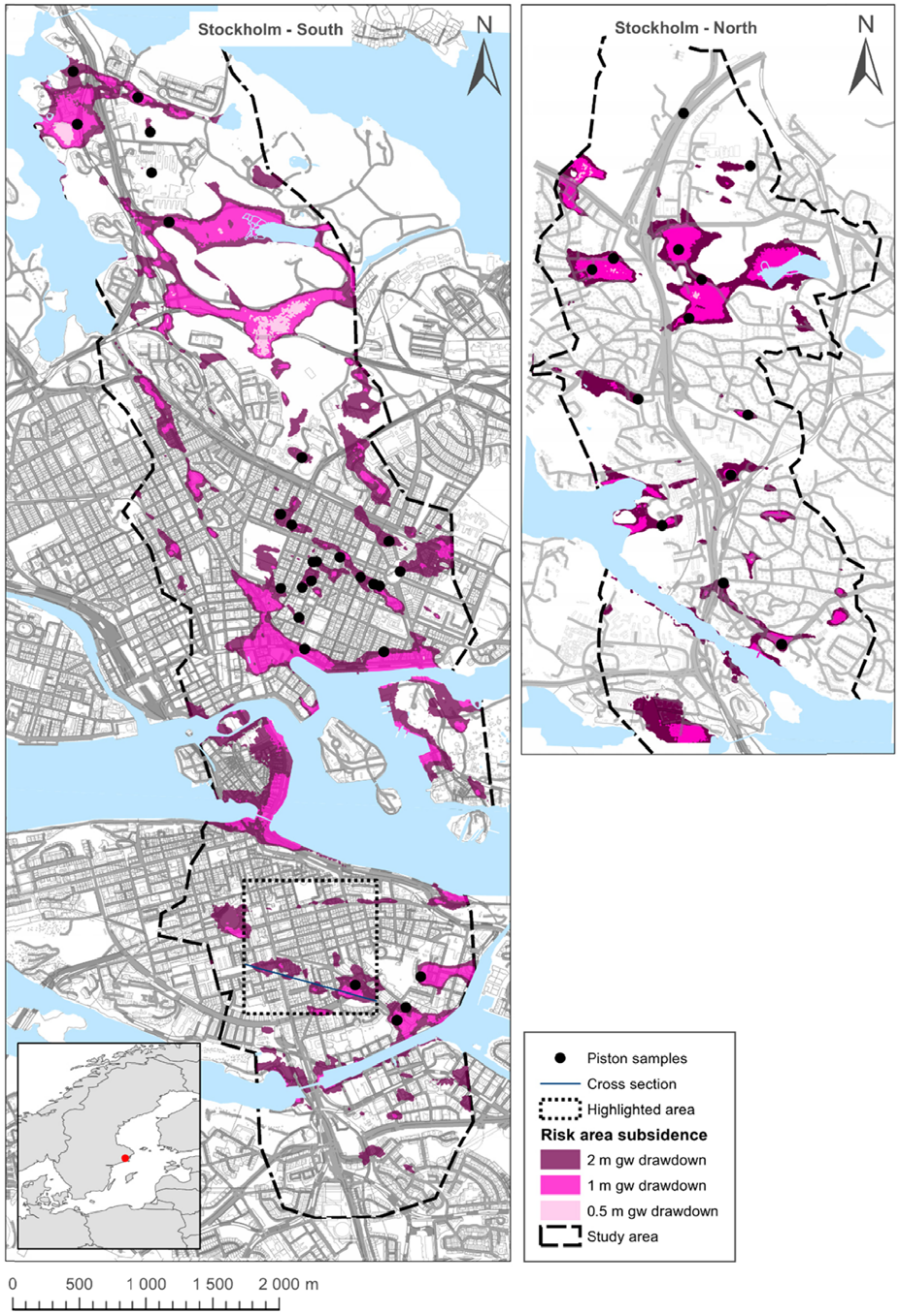
Risk map for subsidence for 0.5 m, 1 m, and 2 m of groundwater drawdown in the confined aquifer. The highlighted area shows the limits for Fig. 10.

From the obtained calculation results, a risk map is produced, distinguishing areas with significant risk from low risk areas for subsidence; see Fig. 11. A risk area is created for each of the three uniform groundwater drawdown scenarios: 0.5 m, 1 m, and 2 m. To exemplify how a risk area can be defined, calculation points where the 95th percentile of the simulations has a land subsidence exceeding 2 cm are selected. Two‐centimeter subsidence has been set as a lower limit for when slight damages can occur in other studies; see, e.g., Son and Cording.^48^ Of course, the probability that the subsidence levels will exceed the 95th percentile value at all locations is much lower. When interpreting the different risk areas, it is important to remember that the subsidence magnitude can vary at different locations within the same area.

To improve usefulness of the risk map, the three uniform scenarios need to be combined with estimations of expected groundwater drawdown at various locations. These estimations are, however, beyond the scope of this article. For the case study, the study area is limited to the maximum expected influence area of groundwater drawdown in the confined aquifer as a result of leakage into the tunnel.

Although the criteria that define the extent of the risk area are reasonable, other percentiles and subsidence limits are possible. When a tolerability criteria is defined in a risk assessment for groundwater drawdown in subsidence‐sensitive areas, it should reflect the acceptance levels of affected stakeholders and norms and regulations in the society.^9^ Based on the tolerability criteria, safety measures can be suggested by means of value of information analysis; see, e.g., Zetterlund *et al*.^49^ For the planned City Link tunnel, the risk maps have been used for communication to stakeholders and authorities in the process for application for permit to drain groundwater in accordance to Swedish legislation.

### 3.6. Sensitivity Analysis

For sensitivity analysis, the Spearman's rank correlation coefficient ρ between each parameter and the simulated subsidence magnitude is calculated; see, e.g., Bedford and Cooke.^50^ More specifically, in a first step the arithmetic mean of the parameter values with depth is calculated. This is repeated at each grid point, yielding a distribution of average values for each realization of the simulation. Finally, the relationship ρ between this distribution and the distribution of subsidence magnitudes is estimated. These steps are carried out for each parameter of interest. A value of ρ close to 1 indicates a strong positive dependence, while a value of ρ close to −1 indicates a strong inverse dependence.

**Figure 12:**
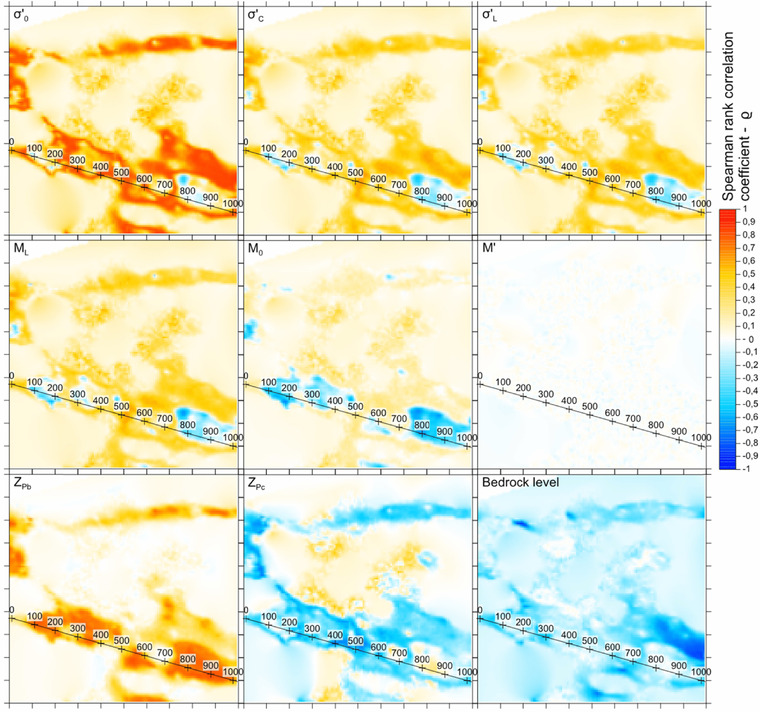
Spearman's rank correlation coefficients for the parameters at different grid points. Each tick mark on the *y*‐ and *x*‐axis represents 100 m distance. The numbered line shows the location for the cross‐section in Fig. 13.

A part of the result of the sensitivity analysis for the 2 m groundwater drawdown simulation is presented in Fig. 12. The results for 0.5 m and 1 m are not included, but show very similar patterns. In addition to the parameters for the subsidence model, two parameters from the soil stratification model are included, *z_pb_* and *z_pc_*. These parameters determine the proportion of clay and coarse‐grained material, respectively.

As described previously, all parameters except for *M′* are dependent on *σ′*_0_. In addition, *σ′*_0_ is dependent on *z_pb_* and *z_pc_* since these parameters determine the clay thickness and its position in the soil profile. This explains the topographic pattern of the sensitivity maps. The map for *M*′ has a zero value across the entire area since the case where Equation (3) takes effect never arises in the example at hand. Moreover, all maps fade to zero correlation at the edges where the clay layer is nonexistent or always above the groundwater level in the confined aquifer. The only parameter persistently positively correlated with land subsidence is the clay proportion, *z_pb_*. Similarly, the bedrock level is always negatively correlated since a deep level results in a thick clay layer. This is consistent with the assumption that thicker clay layers result in larger subsidence.

**Figure 13:**
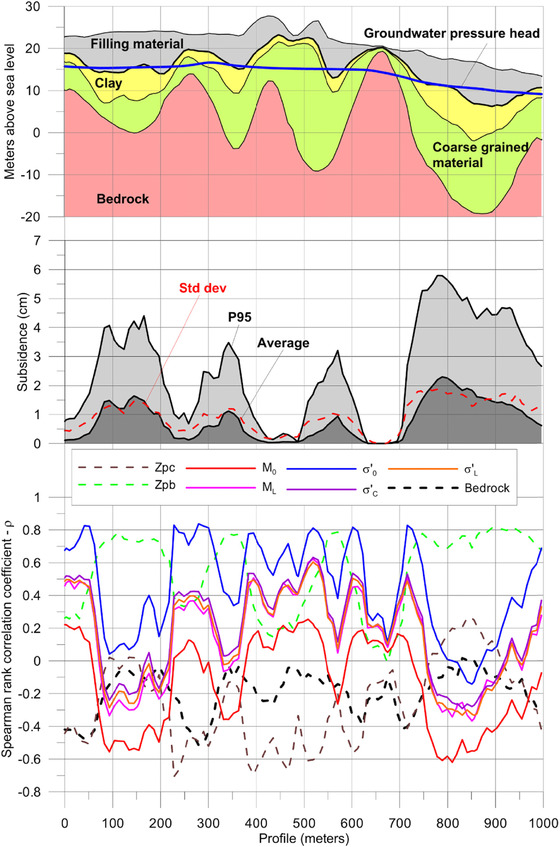
Soil profile with average results of bedrock, soil, and groundwater level in confined aquifer (top). Average and 95th percentile (P95) for simulated subsidence (middle). Spearman's rank correlation for parameters (below). The location of the cross‐section is indicated in Fig. 11.

The result of the rank correlation is explained further from Fig. 13. As seen from the figure, *σ′*_0_, *σ′_c_*, *σ′_L_*, *M_L_*, and *M*_0_ are generally negatively correlated at large subsidence. Large subsidence occurs when the clay layer is relatively thick and in its entirety below the groundwater's piezometric surface. For this situation, the case for Equation (2) takes effect: large subsidence occurs with lower modulus, which is the reason for negative correlation for *M_L_* and *M*_0_. Since *M_L_* and *M*_0_ are positively correlated to the other parameters, *σ′*_0_, *σ′_c_*, and *σ′_L_* also yield negative correlation. An inverse trend with a slightly positive correlation is observed for *z_pc_*. When the clay layer is moved vertically up to the level of the piezometric surface due to a high *z_pc_*, the vertical effective stress is reduced. This also results in smaller modulus.

A gradual shift from this situation takes place when the clay layer goes from below to above the piezometric groundwater level in the confined aquifer (Fig. 13). An inverse situation, with positive correlations for *σ′*_0_, *σ′_c_*, *σ′_L_*, *M_L_*, and *M*_0_ and a negative correlation for *z_pc_*, is observed for smaller subsidence magnitudes. This occurs with thin clay layers that are close to the surface and where the clay layer is often above the groundwater level. With a clay layer above the groundwater table, no subsidence can occur. Since high values for *z_pc_* cause the clay layer to be situated at a higher level, thus above the groundwater surface, this parameter is negatively correlated. The positive correlation for the other parameters is also explained by the same phenomenon. Since this situation is heavily dependent on a clay layer below the groundwater table, *σ′*_0_ also increases when the clay layer has a deeper location. Since *σ′_c_*, *σ′_L_*, *M_L_*, and *M*_0_ are dependent on this parameter, they also show a positive correlation. For this situation, the case according to Equation (1) often takes effect. Although Equation (1) results in larger subsidence with lower *M*_0_, a clay layer below the groundwater surface dominates the situation, which results in larger land subsidence.

### 3.7. Accuracy of Results

The simulation of soil stratification shows good correspondence with a reference data set (30% of all samples were selected and not used for modeling); see Sundell *et al*.^20^ for details. The subsidence simulations, however, cannot be fully validated since they correspond to a future, undesired, scenario. For decision making based on this result, it is important to evaluate if the process representation is sufficiently detailed and accurate to be useful for predicting the governing response in the system.^51^ The novelty in this work is the combination of a probabilistic soil stratification model with a statistical analysis of compression parameters to calculate subsidence with a simple, but nonlinear, method that compares well with individual deterministic predictions performed from single borehole data. Since the soil stratification model is validated and a standardized method for subsidence calculations is used, the forecasted risk areas are expected to be reasonable and useful for the purpose of risk assessment of permanent groundwater drawdowns on a macro scale (1–100 km²).

Although model uncertainties are ignored, such as the assumptions for the pore pressure profile and the use of a simplified numerical model that does not account for creep, our model is able to separate areas with significant risk from low‐risk areas for subsidence. To verify the model on a detailed scale, more advanced models based on the result of individual sampling locations are recommended. We argue that our model combined with such additional refinements gives enough detail to provide relevant decision support on the scale considered here.

Commonly, the constructor of a subsurface project covers the costs for safety measures and not the stakeholders, which suffer from the consequences by subsidence damages (owners of constructions). Due to conflict of interests, it is important to be transparent about assumptions made and the chosen threshold levels for defining risk areas.^52^ Since this method is relatively straightforward in comparison to more advanced numerical models for subsidence, this communication is possible if stakeholders hold basic expertise in geotechnical engineering and statistics in order to understand the fundamentals of the suggested modeling process.

## 4. CONCLUSIONS

This article presents a novel method for combining a probabilistic soil stratification model with statistical analysis of compression parameters for simulation of subsidence on a large area with a simple nonlinear 1D compression model. The result of this simulation is used for creating risk maps where areas with significant risk for subsidence are distinguished from low‐risk areas. The suggested method is useful in cases where the following criteria are fulfilled: when soils demonstrate significant heterogeneity in properties, when data on soil stratification are abundant, when data on soil properties are sparse, when dependencies between soil properties need to be taken into account, when trends along depth for soil properties exist, when the compressible layer is normally or slightly overconsolidated, and when soil layers can be assumed to be continuous.

The mapped risk areas and the result of the sensitivity analysis can be used, together with information on sensitive constructions, for supporting decision making regarding prioritization of further investigations, risk‐reducing measures, and monitoring. In the case for the planned City Link tunnel in Stockholm, the maps have been successfully used for risk communication in legal court in the application for permit to modify groundwater conditions.

For a better understanding of the cause–effect chain from drawdown to subsidence, future research on connecting subsidence models with groundwater models and economic valuation of consequences is recommended. With an improved understanding, the risk for making erroneous decisions on risk‐reducing measures, surveillance, and further investigations can be reduced. Recommended future research includes an improved subsidence model that accounts for time dependencies; to extend the soil stratification and subsidence model with a 3D groundwater model; and to improve the decision making by means of economic valuation of consequences. Although these improvements are suggested, the presented model has in the case study been demonstrated to be a useful decision‐support tool when assessing the risk for groundwater‐drawdown‐induced subsidence on a large scale.

## Supporting information


**Appendix**: Risk mapping of groundwater‐drawdown‐induced land subsidence in heterogeneous soils on large areas
