# Supplementary material for: Risk Mapping of Groundwater‐Drawdown‐Induced Land Subsidence in Heterogeneous Soils on Large Areas
Source: Risk Anal. 2017 Oct 30;39(1):105–24. doi: 10.1111/risa.12890 (PMC7379303; doi:10.1111/risa.12890)

# Appendix

**Risk mapping of groundwater drawdown induced land subsidence in heterogeneous soils on large areas**

Jonas Sundell^a,b,*^, Ezra Haaf^b,c^, Tommy Norberg^d^, Claes Alén^a^, Mats Karlsson^a^, Lars Rosén^a^

^a^ Department of Civil and Environmental Engineering, Chalmers University of Technology, SE-412 96 Gothenburg, Sweden

^b^ COWI AB, Box 12076, SE-402 41 Gothenburg, Sweden

^c^ Department of Earth Sciences, University of Gothenburg, Box 100, SE-405 30 Gothenburg, Sweden

^d^ Department of Mathematical Sciences, Chalmers University of Technology and the University of Gothenburg, SE-412 96 Gothenburg, Sweden

^*^ *Correspondning author*. [jonas.sundell@chalmers.se](mailto:jonas.sundell@chalmers.se)

# Data - clay samples

E and N – coordinates, SWEREF99 18.00.
Depth – depth below surface level [m].
ρ - density [kN/m³].
*σ'_C_* [kPa], *σ'_L_,* [kPa], *M_L_* [kPa]*, M_0_* [kPa], *M'* [dimensionless] – compression parameters, see section 2.3.
*σ'_0_* - effective stress [kPa], see section 2.2.
OCR – over consolidation ratio, *σ'_C_ /σ'_0_.*
DU 1 – Peri-urban greenfield sites, see map.
DU 2 – Sites with light constructions, see map.
DU 3 – Urban areas with heavy constructions, see map.
Area – classed area, see colour code on map, 1 – north most, 5 – south most.

| Borehole | E | N | Depth | ρ | σ´c | σ´L | M0 | ML | M´ | σ´0 | OCR | DU | Area |
| --- | --- | --- | --- | --- | --- | --- | --- | --- | --- | --- | --- | --- | --- |
| 13C120 | 152243 | 6589208 | 2.5 | 17.8 | 79 | 182 | 9750 | 3228 | 6.6 | 34 | 2.34 | 2 | 1 |
| 13C122 | 152745 | 6588815 | 2 | 17.1 | 59 | 91 | 5000 | 444 | 19.1 | 29 | 2.05 | 1 | 1 |
| 13C131 | 152082 | 6586112 | 3.5 | 17.2 | 49 | 90 | 3750 | 613 | 19.7 | 61 | 0.80 | 1 | 2 |
| 13C131 | 152082 | 6586112 | 4.5 | 17.9 | 91 | 137 | 4000 | 1123 | 15.8 | 79 | 1.15 | 1 | 2 |
| 13C133 | 152979 | 6585219 | 4 | 16.9 | 62 | 122 | 3500 | 549 | 15.8 | 45 | 1.39 | 2 | 2 |
| 13C133 | 152979 | 6585219 | 6 | 16.9 | 63 | 96 | 3500 | 582 | 14.4 | 59 | 1.07 | 2 | 2 |
| 13C133 | 152979 | 6585219 | 8 | 17.2 | 79 | 132 | 4000 | 733 | 15.8 | 74 | 1.07 | 2 | 2 |
| 13C133 | 152979 | 6585219 | 10 | 17.8 | 93 | 125 | 4250 | 514 | 16 | 89 | 1.04 | 2 | 2 |
| 13C143 | 151902 | 6587060 | 5.5 | 16.2 | 133 | 202 | 9250 | 1893 | 13.4 | 91 | 1.46 | 2 | 2 |
| 13C143 | 151902 | 6587060 | 6.5 | 18.2 | 151 | 232 | 8000 | 2059 | 15 | 99 | 1.53 | 2 | 2 |
| 13C154 | 152206 | 6588185 | 3 | 16.5 | 40 | 56 | 2375 | - | 13 | 21 | 1.92 | 1 | 1 |
| 13C154 | 152206 | 6588185 | 5 | 17.4 | 83 | 109 | 3750 | - | 16 | 35 | 2.38 | 1 | 1 |
| 13C154 | 152206 | 6588185 | 6 | 17.4 | 96 | 158 | 5000 | 778 | 16.6 | 42 | 2.29 | 1 | 1 |
| 13C166 | 151719 | 6588118 | 2.5 | 17.8 | 53 | 88 | 4250 | 613 | 19.7 | 33 | 1.61 | 1 | 1 |
| 13C166 | 151719 | 6588118 | 3.5 | 16.7 | 53 | 91 | 3500 | 437 | 11.7 | 39 | 1.36 | 1 | 1 |
| 13C166 | 151719 | 6588118 | 5 | 16.8 | 51 | 91 | 3750 | 469 | 16.1 | 48 | 1.06 | 1 | 1 |
| 13C171 | 152599 | 6586491 | 4 | 17.1 | 82 | 107 | 3500 | 550 | 17.7 | 61 | 1.36 | 2 | 2 |
| 13C171 | 152599 | 6586491 | 5.5 | 16.8 | 58 | 89 | 4750 | 600 | 14.7 | 72 | 0.81 | 2 | 2 |
| 13C205 | 153209 | 6583886 | 4 | 16.4 | 44 | 72 | 3750 | 213 | 15.6 | 55 | 0.80 | 1 | 3 |
| 13C205 | 153209 | 6583886 | 6 | 17.9 | 97 | 119 | 4750 | 374 | 17.1 | 69 | 1.40 | 1 | 3 |
| 13C205 | 153209 | 6583886 | 8 | 19.4 | 110 | 162 | 4750 | 786 | 21.5 | 85 | 1.29 | 1 | 3 |
| 13C207 | 153301 | 6583624 | 4 | 17.8 | 115 | 165 | 4750 | 1248 | 14.5 | 61 | 1.87 | 1 | 3 |
| 13C212 | 152752 | 6583684 | 2.5 | 16.9 | 84 | 128 | 5500 | 883 | 12.4 | 32 | 2.63 | 1 | 3 |
| 13C212 | 152752 | 6583684 | 4 | 16 | 61 | 89 | 4000 | 423 | 12.4 | 41 | 1.48 | 1 | 3 |
| 13C212 | 152752 | 6583684 | 6 | 16.1 | 66 | 102 | 4750 | 422 | 10.3 | 54 | 1.23 | 1 | 3 |
| 13C217 | 153441 | 6582948 | 6 | 18.9 | 135 | 220 | 7000 | 2888 | 15.4 | 90 | 1.49 | 1 | 3 |
| 13C217 | 153441 | 6582948 | 8 | 17.5 | 158 | 256 | 8000 | 2910 | 12.2 | 93 | 1.71 | 1 | 3 |
| 13C217 | 153441 | 6582948 | 10 | 19 | 131 | 222 | 8000 | 2682 | 12.7 | 95 | 1.38 | 1 | 3 |
| 13C230 | 152721 | 6584081 | 2 | 16.8 | 59 | 79 | 3500 | 502 | 15 | 35 | 1.68 | 2 | 3 |
| 13C230 | 152721 | 6584081 | 3 | 17.6 | 47 | 98 | 3500 | 664 | 13.7 | 40 | 1.17 | 2 | 3 |
| 13C327 | 155054 | 6579720 | 6 | 17 | 126 | 218 | 8250 | 1385 | 12 | 101 | 1.25 | 3 | 4 |
| 13C327 | 155054 | 6579720 | 7 | 16.7 | 171 | 212 | 8250 | 1176 | 13.6 | 110 | 1.55 | 3 | 4 |
| 13C329 | 154459 | 6579741 | 7.5 | 16.4 | 110 | 139 | 10750 | 598 | 11 | 117 | 0.94 | 3 | 4 |
| 13C329 | 154459 | 6579741 | 9 | 16.3 | 114 | 166 | 6000 | 1109 | 12.4 | 118 | 0.97 | 3 | 4 |
| 13C329 | 154459 | 6579741 | 11 | 19.5 | 98 | 154 | 6000 | 1694 | 18.6 | 123 | 0.80 | 3 | 4 |
| 13C357 | 155175 | 6580324 | 7.5 | 18.5 | 171 | 279 | 8500 | 3007 | 13 | 116 | 1.47 | 3 | 4 |
| 13C365 | 154278 | 6580753 | 4 | 17.8 | 137 | 220 | 5000 | 1562 | 13.9 | 70 | 1.97 | 3 | 4 |
| 13C365 | 154278 | 6580753 | 5.5 | 18.5 | 85 | 144 | 5250 | 1127 | 15.9 | 82 | 1.04 | 3 | 4 |
| 13C365 | 154278 | 6580753 | 7 | 18.3 | 115 | 226 | 9500 | 2069 | 17 | 94 | 1.22 | 3 | 4 |
| 13C369 | 154440 | 6580204 | 4.5 | 18.1 | 141 | 227 | 6500 | 2434 | 12.1 | 86 | 1.65 | 3 | 4 |
| 13C370 | 155021 | 6580214 | 7 | 17.8 | 77 | 133 | 5000 | 2570 | 12.1 | 120 | 0.64 | 3 | 4 |
| 13C370 | 155021 | 6580214 | 8.5 | 18.6 | 65 | 173 | 5250 | 2003 | 14.1 | 137 | 0.47 | 3 | 4 |
| 13C370 | 155021 | 6580214 | 10 | 18.7 | 169 | 239 | 5250 | 2993 | 16.3 | 155 | 1.09 | 3 | 4 |
| 13C371 | 155092 | 6580550 | 7.5 | 17.5 | 157 | 188 | 6750 | 1632 | 15.4 | 129 | 1.22 | 3 | 4 |
| 13C371 | 155092 | 6580550 | 8.5 | 19.5 | 133 | 233 | 6250 | 2745 | 14.7 | 137 | 0.97 | 3 | 4 |
| 13C436 | 155152 | 6576952 | 4 | 16.2 | 78 | 122 | 4750 | 637 | 12.1 | 66 | 1.19 | 3 | 5 |
| 13C436 | 155152 | 6576952 | 6 | 16.3 | 87 | 141 | 4750 | 974 | 14.1 | 77 | 1.12 | 3 | 5 |
| 13C436 | 155152 | 6576952 | 8 | 18 | 114 | 180 | 5000 | 1152 | 13.9 | 91 | 1.25 | 3 | 5 |
| 13C437 | 155218 | 6577049 | 6 | 18 | 170 | 263 | 5750 | 2212 | 14.6 | 107 | 1.58 | 3 | 5 |
| 13C437 | 155218 | 6577049 | 8 | 19.1 | 157 | 226 | 6250 | 4185 | 19.4 | 124 | 1.27 | 3 | 5 |
| 14CW104U | 151558 | 6588033 | 3 | 16.2 | 87 | 115 | 3750 | 871 | 17 | 43 | 2.02 | 2 | 1 |
| 14CW104U | 151558 | 6588033 | 4 | 15.9 | 99 | 123 | 3500 | 686 | 18.9 | 50 | 1.98 | 2 | 1 |
| 14CW301 | 154438 | 6581180 | 4.5 | 17 | 90 | 132 | 5000 | 810 | 15.3 | 76 | 1.18 | 3 | 4 |
| 14CW301 | 154438 | 6581180 | 6 | 17.7 | 105 | 161 | 5000 | 1056 | 13.9 | 77 | 1.36 | 3 | 4 |
| 14CW312U | 154362 | 6580673 | 6.7 | 18.7 | 180 | 341 | 5000 | 1755 | 24.1 | - | - | 3 | 4 |
| 14CW318 | 154521 | 6580397 | 4.5 | 17.2 | 118 | 175 | 4750 | 1294 | 13.4 | - | - | 3 | 4 |
| 14CW318 | 154521 | 6580397 | 6 | 18.7 | * | 234 | 6500 | 4057 | 15.1 | - | - | 3 | 4 |
| 14CW319 | 154547 | 6580399 | 5 | 17.9 | 98 | 200 | 4750 | 2135 | 16.3 | 81 | 1.21 | 3 | 4 |
| 14CW323 | 154979 | 6580228 | 7 | 17.5 | 102 | 189 | 5000 | 1726 | 13.8 | - | - | 3 | 4 |
| 14CW323 | 154979 | 6580228 | 8.5 | 17.7 | 128 | 204 | 5750 | 2164 | 15.8 | - | - | 3 | 4 |
| 14CW324 | 154508 | 6580255 | 4.5 | 17.2 | 65 | 129 | 1100 | 1056 | 14.7 | 71 | 0.92 | 3 | 4 |
| 14CW324 | 154508 | 6580255 | 5.5 | 18.2 | 83 | 136 | 4500 | 783 | 13.9 | 85 | 0.97 | 3 | 4 |
| 14CW327U | 154280 | 6580198 | 3.4 | 17.9 | 104 | 155 | 6000 | 1214 | 14.3 | 63 | 1.64 | 3 | 4 |
| 13C113 | 153857 | 6578487 | 5 | 17.8 | 110 | 140 | 5000 | 827 | 16.6 | 77 | 1.44 | 2 | 2 |
| 13C113 | 153857 | 6578487 | 7 | 17.6 | 96 | 159 | 5250 | 1674 | 15.4 | 96 | 1.00 | 2 | 2 |
| 13C162 | 152377 | 6587957 | 2.5 | 16.3 | 24 | 52 | 2500 | 262 | 13.9 | 18 | 1.36 | 1 | 1 |
| 13C197 | 152283 | 6587668 | 2 | 17.1 | 78 | 162 | 4750 | 1390 | 11.8 | 33 | 2.36 | 1 | 1 |
| 13C197 | 152283 | 6587668 | 3 | 17 | 48 | 70 | 3500 | 267 | 16.2 | 39 | 1.22 | 1 | 1 |
| 13C197 | 152283 | 6587668 | 4 | 16.3 | 61 | 90 | 3750 | 335 | 14.5 | 45 | 1.34 | 1 | 1 |
| 13C353 | 154415 | 6579978 | 4 | 17.3 | 138 | 208 | 6750 | 1447 | 11.6 | 68 | 2.03 | 3 | 4 |
| 13C460 | 155329 | 6577278 | 4 | 17.5 | 120 | 211 | 4250 | 1648 | 15.3 | 70 | 1.71 | 2 | 5 |
| 14C316 | 154722 | 6580431 | 6 | 18.1 | 38 | 59 |  | 744 | 15 | - | - | 3 | 4 |
| 14C322 | 154877 | 6580280 | 7 | 18 | 128 | 192 | 6000 | 1475 | 14.5 | 95 | 1.34 | 3 | 4 |
| 14C112 | 152727 | 6586945 | 3 | 17.7 | 119 | 191 | 5750 | 1374 | 14.5 | 42 | 2.85 | 2 | 2 |
| 14C112 | 152727 | 6586945 | 5 | 18 | 117 | 164 | 5000 | 1063 | 17.7 | 49 | 2.37 | 2 | 2 |
| 14C202 | 153311 | 6583320 | 2.5 | 16.5 | 68 | 116 |  | 1282 | 13 | 43 | 1.58 | 1 | 3 |
| 14C419 | 154836 | 6577216 | 9 | 19.1 | 166 | 227 | 6250 | 4186 | 17.1 | - | - | 3 | 5 |
| 14C323 | 154979 | 6580228 | 7 | 17.8 | 102 | 189 | 5000 | 1726 | 13.8 | 29 | 3.55 | 3 | 4 |
| 14C323 | 154979 | 6580228 | 8.5 | 18 | 128 | 204 | 5750 | 2164 | 15.8 | 36 | 3.56 | 3 | 4 |

# Map – classed areas


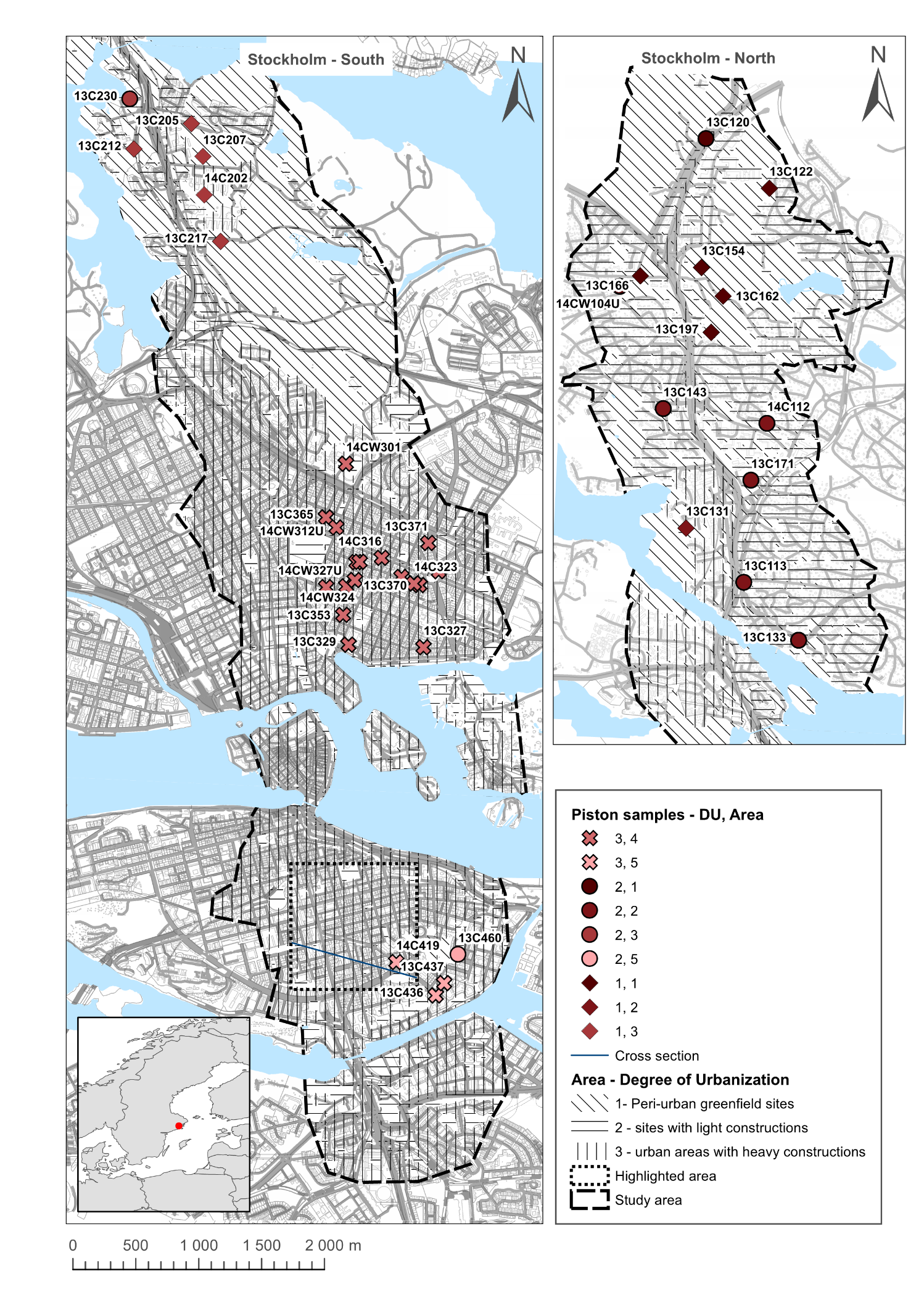


# Variograms – transformed parameters


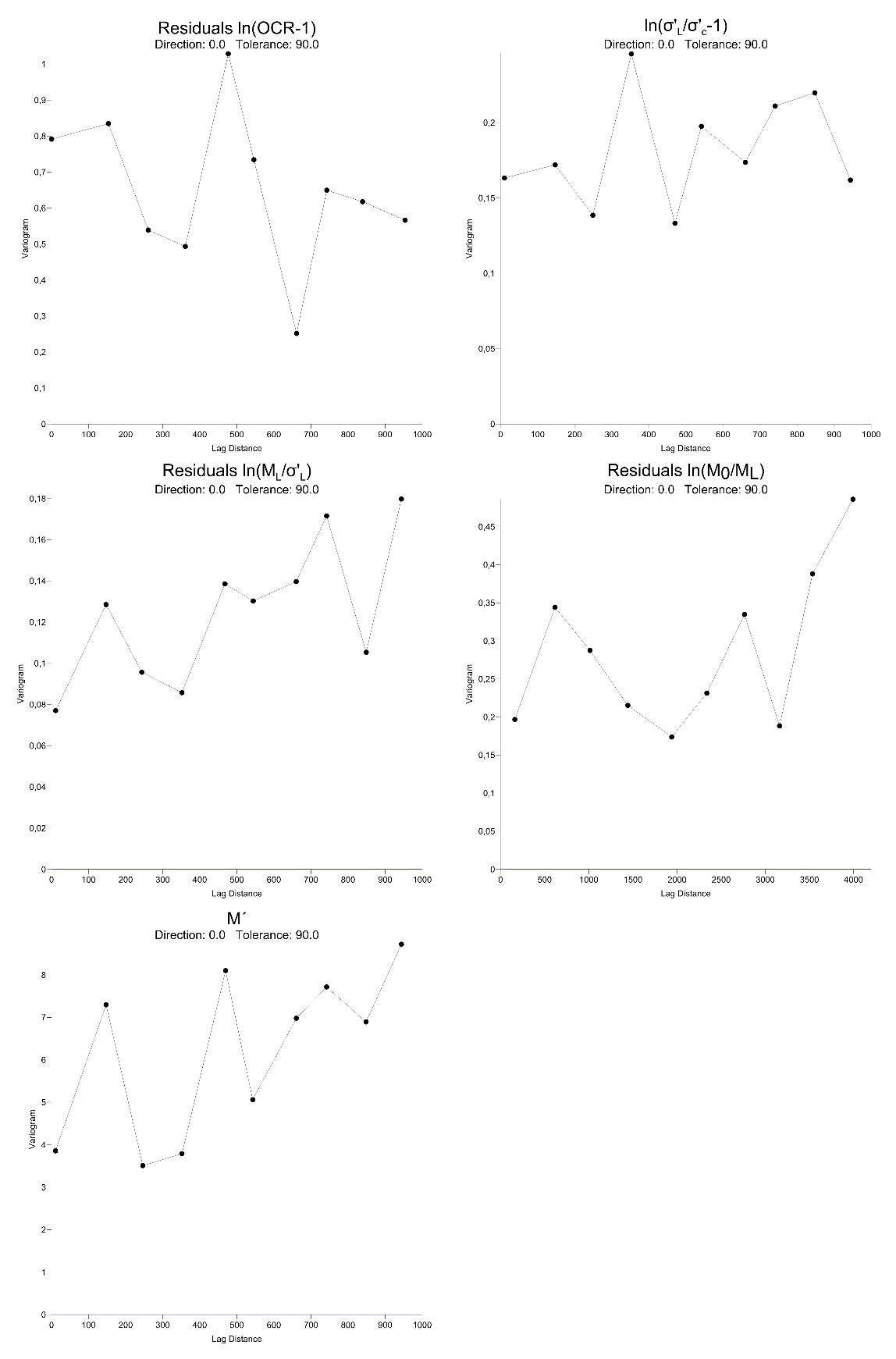

Supplement: Supplementary file 1 — Appendix: Risk mapping of groundwater‐drawdown‐induced land subsidence in heterogeneous soils on large areas [file RISA-39-105-s001.docx]
